# Oblique contractional reactivation of inherited heterogeneities: Cause for arcuate orogens

**DOI:** 10.1002/2016TC004424

**Published:** 2017-03-24

**Authors:** Elisa Calignano, D. Sokoutis, E. Willingshofer, J.‐P. Brun, F. Gueydan, S. Cloetingh

**Affiliations:** ^1^ Faculty of GeosciencesUniversity of UtrechtUtrechtNetherlands; ^2^ Department of GeosciencesUniversity of OsloOsloNorway; ^3^ Géosciences Rennes UMR 6118 CNRSRennes 1 UniversityRennesFrance; ^4^ Géosciences MontpellierUniversité de MontpellierMontpellierFrance

**Keywords:** analog models, oblique shortening, tectonic inheritance, strong domain, Ouachita orogen

## Abstract

We use lithospheric‐scale analog models to study the reactivation of pre‐existing heterogeneities under oblique shortening and its relation to the origin of arcuate orogens. Reactivation of inherited rheological heterogeneities is an important mechanism for localization of deformation in compressional settings and consequent initiation of contractional structures during orogenesis. However, the presence of an inherited heterogeneity in the lithosphere is in itself not sufficient for its reactivation once the continental lithosphere is shortened. The heterogeneity orientation is important in determining if reactivation occurs and to which extent. This study aims at giving insights on this process by means of analog experiments in which a linear lithospheric heterogeneity trends with various angles to the shortening direction. In particular, the key parameter investigated is the orientation (angle *α*) of a strong domain (SD) with respect to the shortening direction. Experimental results show that angles *α* ≥ 75° (high obliquity) allow for reactivation along the entire SD and the development of a linear orogen. For *α* ≤ 60° (low obliquity) the models are characterized by the development of an arcuate orogen, with the SD remaining partially non‐reactivated. These results provide a new mechanism for the origin of some arcuate orogens, in which orocline formation was not driven by indentation or subduction processes, but by oblique shortening of inherited heterogeneities, as exemplified by the Ouachita orogen of the southern U.S.

## Key Points

- Analog models investigate oblique shortening of a heterogeneous lithosphere
- Obliquity of a strong domain determines degree of strain localization
- An arcuate orogen forms for certain obliquity angles of the strong domain

## 1 Introduction

Curved orogenic belts are rather common structures in contractional settings, and their origin is attributed to various mechanisms. On the basis of the relationship between orientation of thrusts and folds and vertical axis rotations, arcuate orogens can be primary or secondary (oroclines) or develop their curvature progressively during shortening [*Weil and Sussman*, 2004]. Among the proposed mechanisms, orogen‐parallel compression has been suggested to explain oroclines [*Johnston et al*., 2013; *Weil et al*., 2013], while slab rollback and associated slab tearing was proposed to be at the origin of most progressive arcs in the Mediterranean region and west Pacific Ocean [*Faccenna et al*., 2004; *Rosenbaum and Lister*, 2004; *Royden*, 1993]. The curved shape of primary arcs is strongly influenced by pre‐existing heterogeneities of the colliding continental blocks, e.g., an irregular margin, the shape of an indenter, or lateral variation in sediment thickness and/or rheology [*Macedo and Marshak*, 1999]. Altogether, tectonic inheritance is an important factor controlling the location and structural trend of orogenic systems [*Audet and Bürgmann*, 2011]. The presence of lateral strength contrasts in the lithosphere controls strain localization and thus the initiation and subsequent evolution of mountain belts [*Vauchez et al*., 1998]. In particular, previous modeling studies have shown that the orientation of pre‐existing heterogeneities with respect to the applied shortening direction determines the degree of their reactivation [*Amilibia Cabeza et al*., 2005; *Bonini et al*., 2012; *Brun and Nalpas*, 1996]. However, most of these studies limit their investigation to the scale of the crust. The models presented in *Calignano et al*. [2015] illustrated how the initiation and subsequent evolution of orogens in continental compressional settings were the result of the presence of a high‐strength localizing lithospheric mantle (independent from the presence of crustal heterogeneities) and localization at the margins of a stronger lithospheric domain in the crust. Therefore, it is important to test the effect of the orientation of pre‐existing heterogeneities at a lithospheric scale. In the present paper, we use lithospheric‐scale analog models to study the reactivation of a tabular heterogeneity in the continental lithosphere undergoing oblique shortening and we demonstrate that such kinematic and rheological conditions favor the development of arcuate orogens.

## 2 Experimental Setup

### 2.1 Initial Geometry

**Figure 1:**
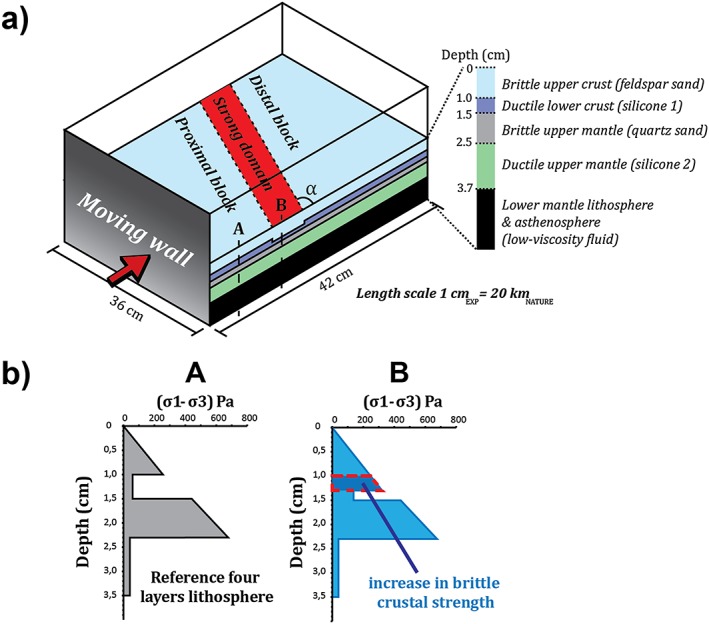
(a) Experimental setup: four‐layer lithosphere modeled with alternating frictional/viscous materials (sand and silicon putties) and resting on a low‐viscosity, high‐density fluid that guarantees isostatic compensation. A stronger domain (SD: thicker brittle upper crust) striking at an angle *α* to the shortening direction (red arrow) is present in all models. Model shortening occurs by a moving wall displacing at constant rate inside the Plexiglas box. (b) Representative experimental strength profiles showing lateral variation in lithospheric strength at initial stage of deformation.

The initial geometry of the experiments is shown in Figure 1, and the geometric and kinematic parameters are listed in Table 1.

**Table 1:** Geometrical and Kinematical Parameters^a^

Experiment	Width (cm)	Length (cm)	*h*_UC_ (cm)	*h*_LC_ (cm)	*h*_BUM_ (cm)	*h*_DUM_ (cm)	Obliquity Angle *α* (°)	Velocity (cm/h)
1	36.00	42.0	1.0–1.3	0.5–0.2	1.0	1.3	90	1.0
2	36.0	42.0	1.0–1.3	0.5–0.2	1.0	1.3	80	1.0
3	36.0	42.0	1.0–1.3	0.5–0.2	1.0	1.3	75	1.0
4	36.0	42.0	1.0–1.3	0.5–0.2	1.0	1.3	60	1.0
5	36.0	42.0	1.0–1.3	0.5–0.2	1.0	1.3	45	1.0

a The values for the thickness of upper crust (*h*_UC_) and lower crust (*h*_LC_) refer to the proximal/distal block (left) and central block (right).

The experiments are scaled such that 1 cm equals 20 km in nature, thus covering an area equivalent to 840 km × 720 km.

In the central part of all models, the thickness of the brittle upper crust is increased in order to simulate the presence of a stronger domain (SD) along a band trending at an angle *α* to the shortening (*α* = 90°, 80°, 75°, 60°, and 45°).

This setup simulates the deformation of an overall stiff, yet stratified continental lithosphere containing a stronger domain, representing an area of low geothermal gradient, which is found in cases where the lower crust is mafic in composition or when continental rifts are thermally equilibrated [*Calignano et al*., 2015].

Lateral strength variations are common in continental lithosphere and are mostly related to changes in composition or thermal structure affecting the crust or the mantle or both layers. For example, the strength of the crust can increase after melt extraction, leaving behind a dehydrated residue, or in the presence of high‐viscosity mafic lower crust, which is often associated with a low concentration of heat‐producing radiogenic elements [*Bürgmann and Dresen*, 2008]. Similar to the experiments presented in *Calignano et al*. [2015], the experiments of this study have been performed against the background of a thinned lithosphere, where the process of rifting exhumed the mantle lithosphere to shallow depth [*McKenzie*, 1978]. Subsequent cooling may increase the strength of the thinned lithosphere beyond that of the unstretched one [*Buiter et al*., 2009; *Cloetingh et al*., 2008; *Leroy et al*., 2008; *McKenzie*, 1978]. As such, (failed) continental rift are among the most important sites of lateral strength contrasts in the continental lithosphere [*Vauchez et al*., 1998]. We approach the strong cold/old rift by implementing a linear zone of high strength, where the crustal strength is distinctly increased with respect to the surrounding lithosphere (Figure 1). As a result, the thick brittle crust is strongly coupled to the strong mantle lithosphere within this domain, creating a local increase in bulk lithospheric strength similar to a situation where the Moho would be at shallow crustal level [e.g., *Jammes and Huismans*, 2012].

### 2.2 Rheology

The reference model lithosphere consists of four layers with strength maxima in the brittle upper crust and brittle upper mantle (Figure 1a), representing regions of intermediate‐to‐low geothermal gradient [*Gueydan et al*., 2008], such as a thermally re‐equilibrated rift. If, during rifting, the high geothermal gradient weakens the lithospheric mantle and it could be represented by a three‐layer system, then later thermal relaxation restores its strength profile to a four‐layer type with an upper high‐strength lithospheric mantle. The calculated strength envelopes shown in Figure 1b are representative for the very initial stage of deformation (see Appendix A for strength profiles). The four‐layer (brittle upper crust, ductile lower crust, brittle lithospheric mantle, and ductile lithospheric mantle) model lithosphere is floating on the model asthenosphere, a low‐viscosity and high‐density fluid made of a mixture of polytungstate and glycerol, ensuring isostatic compensation (Figure 1a). Dry feldspar sand and dry quartz sand, exhibiting Mohr‐Coulomb type behavior, are used to simulate the brittle behavior of the upper crust and the upper lithospheric mantle, respectively. Densities and coefficients of friction for these granular materials are given in Table 2. Different colored sand layers help the visualization of brittle structures in cross section. Ductile behavior of the lower crust and the lithospheric mantle is accounted for through mixtures of Rhodorsil Gomme CSIR (Rhône Poulenc, France)‐type silicon putty, quartz sand as filler to calibrate densities, whereas oleic acid was added to modulate viscosity. Both ductile materials show viscous quasi‐Newtonian behavior (Table 2). The density of the lithospheric mantle is in all experiments lower than the asthenospheric fluid, so that subduction is impeded.

**Table 2:** Rheological Parameters for the Analog Materials Used in the Experiments^a^

Layer	Material	Density *ρ* (kg m^−3^)	Coeff. Friction *μ*	Cohesion *C* (Pa)	Stress Exponent *n*	Material Constant *A*
Brittle upper crust	Dry feldspar sand	1300	0.4–0.7	15–35		
Ductile lower crust	Silicon 1	1400			1.16	1.00 × 10^−5^
Brittle lithospheric mantle	Dry quartz sand	1500	0.6	30–70		
Ductile lithospheric mantle	Silicon 2	1578			1.06	1.00 × 10^−5^
Lower lithosphere + asthenosphere	Sodium polytungstate + glycerol	1600				

a The values of parameters *n*, *A*, and effective viscosity have been determined with a Cony‐cylindrical viscometer at a room temperature of 20 ± 2°C.

### 2.3 Experimental Procedure

The models are built inside a 36 cm × 50 cm × 15 cm box of transparent Plexiglas. All experiments were shortened by 8 cm at a constant velocity of 1.0 cm h^−1^, which scales to a range of velocities of approximately 4–7 mm/yr in nature. Experiments are performed in normal gravity field, and thin glass plates along the long side of the tank serve to reduce boundary effects due to friction. Top‐view digital imagery and laser scanning at regular time intervals are used to monitor deformation. Based on the scanner data, digital elevation models (DEMs) have been calculated for a detailed analysis of the topographic evolution. At the end of an experiment the model is soaked in water, frozen, and cut in cross sections parallel to the convergence direction, allowing for the visualization of the final model geometry.

### 2.4 Scaling

Yet we seek to demonstrate the relevance of the experimental results for the Ouachita orocline, the experiments are generic in nature and thus potentially also applicable to other regions. As such, we scaled the experiments to average convergence rates and ductile layer viscosities in nature. The experiments are built with a length scale factor of 5.0 × 10^−7^ so that 1 cm in the model corresponds to 20 km in nature.

Scaling is dome on the principle of geometrical, rheological, dynamical, and kinematic similarity [*Davy and Cobbold*, 1991; *Ramberg*, 1981; *Weijermars and Schmeling*, 1986], using dimensionless rations of gravitational to viscous forces (Table 3) as expressed in the Ramberg number (*R_m_*) for ductile layers:

(1) $$R_{m}=\frac{\rho gh_{d}}{{\left(\sigma_{1}-\sigma_{3}\right)}_{\text{viscous}}}$$

where *ρ* and *h* are respectively the density and thickness of the ductile layer, *g* is the acceleration due to gravity (*g* = 9.81 m/s^2^), and (*σ*_1_ − *σ*_3_)_viscous_ is the strength for ductile materials with power law‐type behavior (see Appendix A). Similarly, we used the Smoluchowski number (*S_m_*) defined by *Ramberg* [1981] to control dynamic similarity for brittle materials as follows:

(2) $$s_{m}=\frac{\rho gh}{c+\text{μρgh}}$$

where *ρ*, *h*, *c*, and *μ* are respectively the density, thickness, cohesion, and coefficient of friction of the brittle layer.

**Table 3:** Scaling Parameters^a^

Layer	Density *ρ* (kg m^−3^)	Thickness *h* (m)	Effective Viscosity *η* (Pa s)	Strain Rate (s^−1^)	*S_m_*	*R_m_*	*R_e_*
Brittle upper crust model	1300	0.01			7.5		
Brittle upper crust nature	2700	20,000			5.7		
Ductile lower crust model	1352	0.005	5.75 × 10^4^	5.56 × 10^−4^		1.08	3.38 × 10^−10^
Ductile lower crust nature	2900	10,000	1.00 × 10^22^	1.0 × 10^−15^		1.79	3.68 × 10^−25^
Brittle lithospheric mantle model	1500	0.01			4.2		
Brittle lithospheric mantle nature	3000	10,000			6.1		
Ductile lithospheric mantle model	1578	0.013	8.37 × 10^4^	2.14 × 10^−4^		5.60	6.81 × 10^−10^
Ductile lithospheric mantle nature	3300	26,000	2.00 × 10^22^	1.0 × 10^−15^		6.90	1.19 × 10^−20^

a For the *S_m_* the lower values of cohesion and coefficient of friction for analog materials are considered.

The dynamic similarity requires that other non‐dimensional numbers involving inertial forces are equivalent in model and nature, such as for the Reynolds number, *R_e_* (ratio of inertial forces to viscous forces [*Ramberg*, 1981]):

(3) $$R_{e}=\frac{\rho vh}{\eta }$$

The experiments presented in this paper fulfill the requirement of *R_e_* ≪ 1 (Table 3).

### 2.5 Simplifications

Scaled analog models are simplified representations of natural processes.

Our experiments aim at simulating shortening of a thermally equilibrated continental lithosphere. As such, we are confident that representing the ductile behavior of the lower crust and lithospheric mantle with uniform viscous materials is an acceptable first‐order approximation [*Davy and Cobbold*, 1991], although ductile behavior in nature is strongly dependent on temperature and consequently varies with depth [*Ranalli and Murphy*, 1987]. Furthermore, we study the evolution of topography in the absence of surface processes (erosion, transport, and deposition of sediments). Although the study of *Burov and Toussaint* [2007] has shown that these processes lead to stress redistribution and influence the time evolution and localization of deformation, we argue that they are of secondary importance for the evolution of comparably small convergent zones within intraplate compressional settings as addressed in this contribution.

## 3 Experimental Results

### 3.1 Experiment 1 – SD at 90°

**Figure 2:**
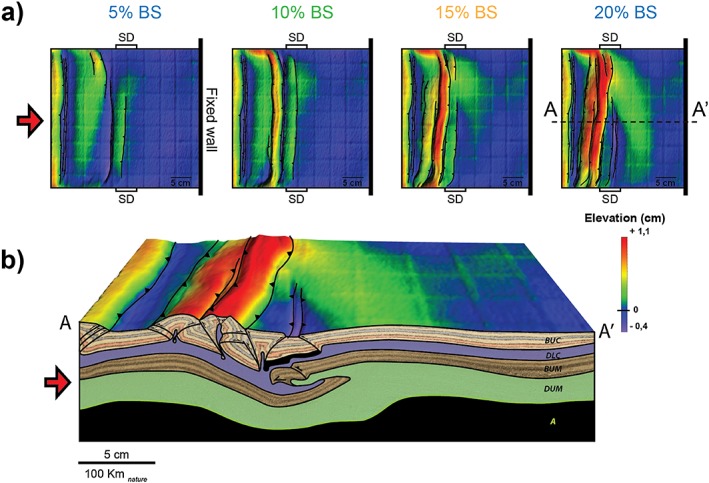
Modeling results for experiment 1: *α* = 90°. (a) Evolution of deformation and associated topography shown through DEMs at 5%, 10%, 15%, and 20% bulk shortening. (b) Final geometry and interpreted structures after 20% bulk shortening illustrated by a representative cross section (A‐A′) and DEM. The thick black line in the upper crust indicates the position of the SD.

After 5% bulk shortening, a pop‐up nucleates at the proximal margin of the strong central block (Figure 2a). The pop‐up is defined by a major thrust and an associated back thrust. Subsequent shortening results in the migration of deformation in the upper crust of the proximal block, toward the moving wall. By 15% bulk shortening, three pop‐up structures delimiting push‐down triangular blocks can be recognized from the DEMs and related topographic profile (Figure 2b). At the end of the experiment the second and third pop‐ups have merged into a prominent uplifted belt, while the first pop‐up shows lower elevation. Uplift of the pop‐up structures is accompanied by normal faulting above rising lower crustal material. During the latest stages of deformation, a minor thrust nucleates at the distal margin of the strong central block that becomes progressively pushed down.

The cross section in Figure 2b shows an asymmetric final geometry of the deep lithospheric structure, characterized by underthrusting of the proximal block under the strong domain and distal block. Brittle failure occurs in the upper lithospheric mantle, and it is localized at the proximal margin of the strong block. Deformation is characterized by strong decoupling between crust and mantle (Figure 2b). Numerous faults bound pop‐up and pop‐down structures in the brittle upper crust. Here deformation propagates progressively from the strong domain toward the moving wall. In contrast, shortening in the upper brittle mantle is accommodated by a single major thrust. The downward movement of the proximal block is accommodated by thinning of the ductile lower lithospheric mantle in the central part of the experiment and associated thickening in correspondence of the distal block.

### 3.2 Experiment 2 – SD at 80°

**Figure 3:**
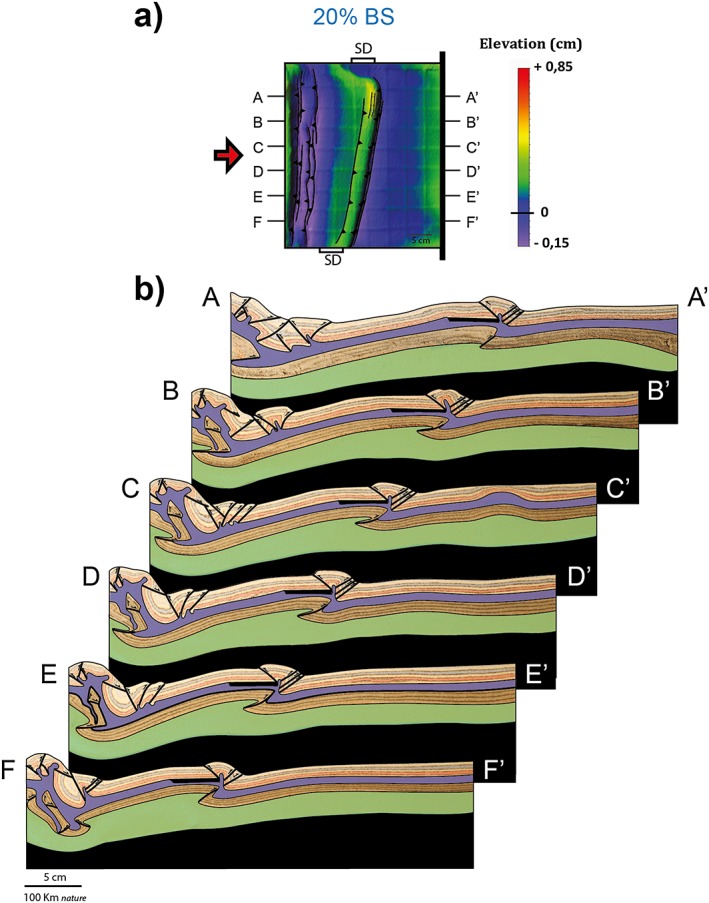
Modeling results for experiment 2: *α* = 80°. (a) Topography 20% bulk shortening. (b) Final geometry and interpreted structures after 20% bulk shortening illustrated by representative cross sections (A‐A′ to F‐F′). The thick black line in the upper crust indicates the position of the SD.

Taking as a reference model experiment 1, where the SD was perpendicular to the shortening direction, the obliquity of the heterogeneity is first decreased by 10°. Most of the shortening during experiment 2 was accommodated by thrusts close to the moving wall, probably due to construction imperfections. However, despite a delay in the appearance of upper crustal faults, the final configuration of the model is in line with the results of the other experiments. For this reason the experiment is described with only the final DEM (Figure 3a). At about 15% bulk shortening, a pop‐up nucleates at the distal boundary of the SD. With ongoing shortening, deformation propagates toward the fixed back wall, as testified by the development of other thrusts to the right of the pop‐up. Eventually, the crustal structure is rather simple, with a linear thrust system following the initial trend of the SD. The cross sections in Figure 3b reveal the presence of a displacement surface in the brittle lithospheric mantle in correspondence of the crustal wedge. Experiment 2 has the same rheological layering and has been shortened with the same velocity as experiment 1, but the SD is now oblique to the shortening direction. Experiment 1 was characterized by localization of upper crustal structures at the proximal margin of the SD and a thrust in the brittle lithospheric mantle dipping toward the fixed back wall (Figure 2). Experiment 2 demonstrates that a slight deviation from the perpendicular case is enough to shift the localization of crustal deformation from the proximal to the distal margin of the SD and reverse the dip of the mantle displacement surface (Figure 3).

### 3.3 Experiment 3 – SD at 75°

**Figure 4:**
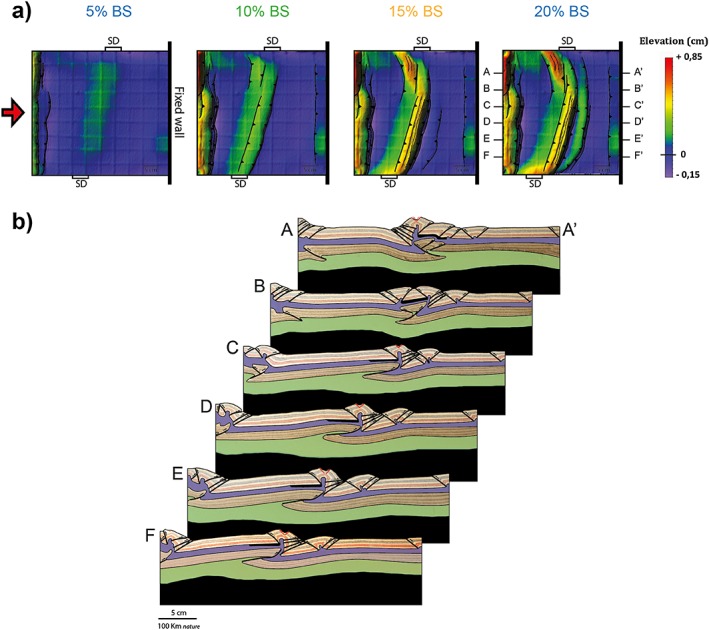
Modeling results for experiment 3: *α* = 75°. (a) Evolution of deformation and associated topography shown through DEMs at 5%, 10%, 15%, and 20% bulk shortening. (b) Final geometry and interpreted structures after 20% bulk shortening illustrated by representative cross sections (A‐A′ to F‐F′). The thick black line in the upper crust indicates the position of the SD. Reverse faults are in black, while normal faults are in red.

In experiment 3 the obliquity of the SD to the shortening direction is further decreased to 75°. The early stages of applied shortening are characterized by uplift in the central part of the experiment, with a trend reflecting the orientation of the SD. Between 5% and 10% bulk shortening, thrust faults develop at the boundaries of the SD (Figure 4a). Different from experiment 2, a pop‐up structure nucleates at the distal margin of the SD, striking from the bottom of the model up to about 65% of its length, while on the remaining 35% of the SD, the pop‐up is localized at the proximal margin, similar to the 90° case. With ongoing shortening activity along the conjugate thrusts, bounding the pop‐up continues as testified by continuous uplift and eventually appearance of minor normal faults on the crest of these structures. Contemporaneously, new thrusts form to the left of the pop‐up, where this is located at the distal boundary of the SD, and to the right when the pop‐up is localized at the proximal margin of the SD. The final top view architecture reveals a clear difference with respect to experiments with 80°: the thrust system deviates from the linear trend observed in the previous experiments to attain a more complex and somewhat curved trend. The cross sections of Figure 4b reveal a complex pattern of thrusts in the upper brittle crust and a single displacement surface in the high‐strength lithospheric mantle. Where the first crustal pop‐up is localized at the proximal margin of the SD, the thrust in the mantle dips toward the moving wall (section A, Figure 4b), while where it is localized at the opposite margin, it shows opposite dip (sections C–F, Figure 4b). The complex geometry observed in section B (Figure 4b) is due to the fact that it crosses a transfer zone between the two thrust systems.

### 3.4 Experiment 4 – SD at 60°

**Figure 5:**
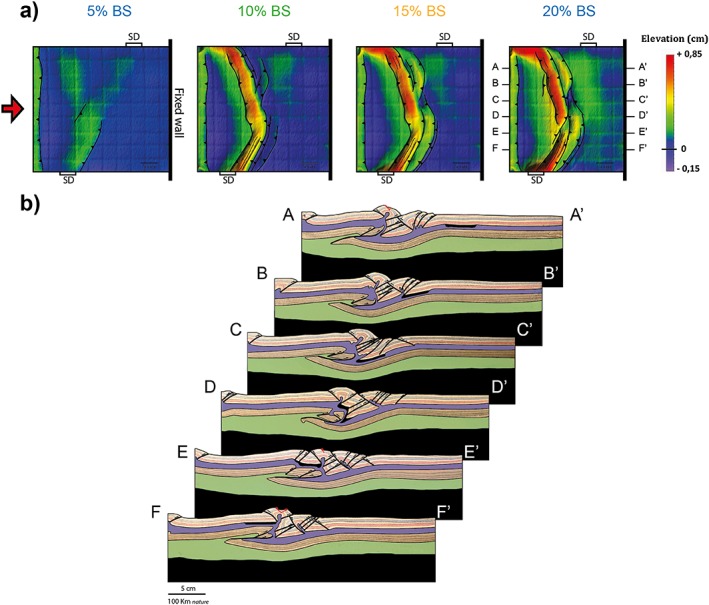
Modeling results for experiment 4: *α* = 60°. (a) Evolution of deformation and associated topography shown through DEMs at 5%, 10%, 15%, and 20% bulk shortening. (b) Final geometry and interpreted structures after 20% bulk shortening illustrated by representative cross sections (A‐A′ to F‐F′). The thick black line in the upper crust indicates the position of the SD. Reverse faults are in black, while normal faults are in red.

An uplifted belt corresponding to the SD forms in the center of the model soon after shortening is applied. At about 5% bulk shortening, strain is partitioned in two distinct uplifted domains: the first in correspondence of the SD spanning from the bottom of the box up to about half the model width, while the second one trending perpendicular to the shortening direction and located at about 12 cm from the moving wall, from the top of the box down to half the model width (Figure 5a). With ongoing shortening, a pop‐up structure develops at the location of the described uplift. The conjugate thrusts are continuous, but their trend changes along the belt strike. In the bottom part of the model the distal SD margin is reactivated and the thrust system attains the same trend as the SD, while in the top part of the model, thrusts trend almost perpendicular to the shortening direction. Thus, with respect to the previous experiment, the length of the reactivated part of the SD decreased from 65% to about 42% of its initial length. Deformation continues to propagate toward the fixed back wall with a series of closely spaced thrusts and a new pop‐up to the right of the first one. The new structures display in top view a curved trend. During the latest stages of deformation, out‐of‐sequence thrusts with a trend parallel to the SD margins become active in the internal part of the wedge, causing subsidence of the first pop‐up at the location where the trend shifts from parallel to the SD to perpendicular to the shortening direction. The cross sections in Figure 5b show the different position at depth of the SD along the width of the model. Similar to experiment 3, a single displacement surface in the high‐strength mantle corresponds to a complex wedge in the brittle crust, due to the decoupling along the ductile lower crust. Sections A‐A′ and B‐B′ cut through the part of the thrust system trending perpendicular to the shortening direction. In particular, the SD is completely undisturbed in section A‐A′, where it lies in the foreland of the thrust system, while in section B‐B′, the latest thrust nucleates at its proximal margin. Sections C‐C′, D‐D′, and E‐E′ are representative for the transition between the trends of the thrust system. Here the SD is deformed and at places buried under the thrust wedge (section D‐D′). Finally, section F‐F′ crosses the part of the thrust system trending parallel to the SD. In this case the distal margin of the SD efficiently localized the first pop‐up structure.

### 3.5 Experiment 5 – SD at 45°

**Figure 6:**
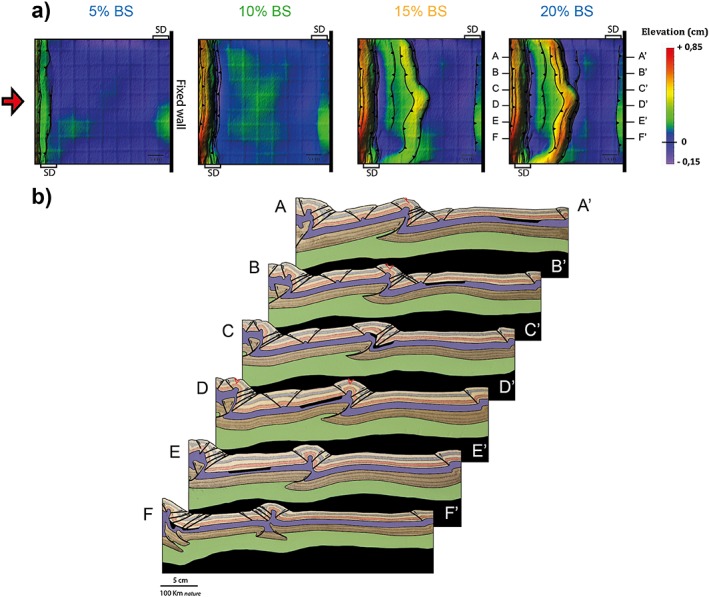
Modeling results for experiment 5: *α* = 45°. (a) Evolution of deformation and associated topography shown through DEMs at 5%, 10%, 15%, and 20% bulk shortening. (b) Final geometry and interpreted structures after 20% bulk shortening illustrated by representative cross sections (A‐A′ to F‐F′). The thick black line in the upper crust indicates the position of the SD. Reverse faults are in black, while normal faults are in red.

The last experiment tests an obliquity angle of 45° between the SD and the shortening direction. Similar to experiment 4, as soon as shortening is applied, the first response of the lithosphere model is localized uplift. At 10% bulk shortening, three regions of uplift can be distinguished on the base of the trend of their axis (Figure 6a). Differently from the previous experiment, the uplift with trend parallel to the SD is in this model localized in the center of the SD. Two regions of uplift with trend perpendicular to the shortening direction can be recognized: one, similar to the previous experiment, is located at about 14 cm from the moving wall and strikes from the top of the model down to about half width; the second strikes from half the model width to the bottom of the model and is located at a distance of 15 cm from the moving wall. With ongoing shortening, a pop‐up will eventually localize along the uplift belts, with a final curved trend in top view. Thus, with respect to experiment 4, the portion of reactivated SD is shorter (about 20% of the initial length) and located in the center of the model. The latest stages of deformation are characterized by the propagation of deformation toward the back wall in the top part of the model. Again, the new thrust displays a curved trend, partially following the SD margin. The different location of the SD with respect to the thrust system is clearly shown in the cross sections (Figure 6b). Sections A‐A′ and E‐E′ represent a situation where the SD is not reactivated and remained undeformed in the distal or proximal lithospheric blocks, respectively. In section F‐F′ the SD is also not involved in the main thrust system, but its vicinity to the moving wall allows for its deformation. In section B‐B′ the wedge is located to the left of the SD, but the latest thrusts reactivated at its proximal margin. Section C‐C′ crosses the transition between the part of the thrust system with trend parallel to the SD and the one perpendicular to the shortening direction. Similar to the previous experiment, in this transition zone, the SD is deformed. Section D‐D′ is representative for the reactivated segment of the SD, with the main pop‐up localized at its distal margin.

## 4 Discussion

### 4.1 Strain Localization at the Boundaries of Strong Lithospheric Domains

Initiation and subsequent evolution of orogenic systems strongly depend on the rheological structure of the continental lithosphere and, in particular, on the presence of lateral variations in the lithosphere strength, which contribute to strain localization [*Burov*, 2011; *Ziegler et al*., 1998]. Similar to previous modeling studies [*Davy and Cobbold*, 1991; *Toussaint et al*., 2004], the experiments presented in this paper show that the shortening of a four‐layer continental lithosphere (i.e., with a high‐strength lithospheric mantle) favors the development of intracontinental subduction. The main thrust in the mantle localizes at a characteristic distance (around 12 cm in all experiments) from the moving wall that likely depends on the integrated strength of the continental lithosphere, which determines the wavelength of lithosphere buckling [*Cloetingh et al*., 1999]. In all experiments, model surface effectively displays a broad low amplitude syncline depression between the mobile wall and the thrust belt. The development of a thrust belt in the brittle upper crust is strongly controlled by decoupling along the ductile crust, as shown in the previous experiments of *Willingshofer et al*. [2013].

The location of the displacement surface in the lithospheric mantle primarily depends on the model rheological layering. However, the experimental results emphasize that the presence of strength heterogeneities contributes to its localization, as the SD boundary that corresponds to lateral strength gradient controls the localization of the first thrust in the brittle crust. Reactivation of pre‐existing heterogeneities is rather common in many compressional settings [*Ziegler et al*., 1998]. Previous analog models, applied to the Central Alps collision, investigated the effects of lateral variation in crustal thickness on large‐scale buckling [*Burg et al*., 2002], showing, similar to our models, that crustal buckling can play an important role in continental collision. Moreover, in models with blocks of variable crustal thickness, a narrow anticline developed in the thin‐crust block at the boundary between two thick‐crust blocks. This structural pattern directly compares to the narrow belt located at the SD boundary in our experiment 1 (Figure 2). However, contrary to our models, a continental subduction did not develop in these models, due to the absence of a brittle lithospheric mantle.

### 4.2 Influence of the Obliquity of the Strong Domain

**Figure 7:**
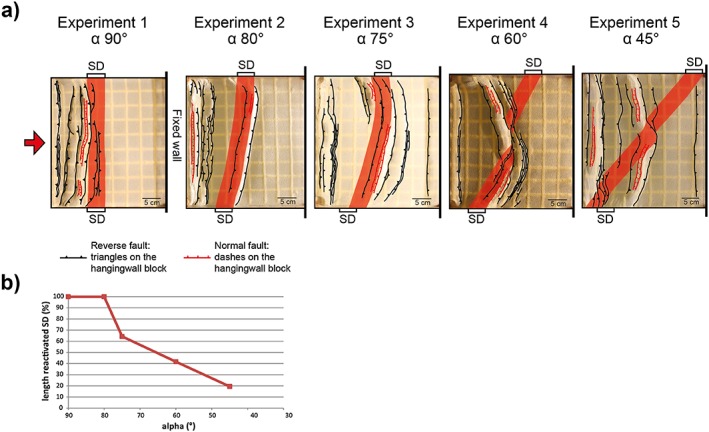
(a) Final top view photograph with fault traces of models, for five obliquity angles (90° ≤ *α* ≤ 45°). The red areas indicate the position of the SD at the end of deformation. (b) Plot showing the dependency of reactivated length of the SD to the obliquity angle *α*.

The experimental results are described based on the top views of final structures for different obliquity angles *α* between the SD and the shortening direction (Figure 7a). In all experiments, soon after the onset of shortening, pop‐up structures developed in the brittle upper crust. Models with *α* = 90° and 80° display pronounced strain localization against one of the boundaries of the SD, with the development of a linear thrust belt since the early stages of deformation. At *α* = 90°, deformation localized at the proximal margin of the SD (i.e., toward the moving wall). With ongoing shortening, pop‐up structures progressively migrated toward the moving wall, thanks to effective decoupling along the ductile lower crust. On the contrary, at *α* = 80°, a single pop‐up localized at the distal margin of the SD. With decreasing obliquity, a progressively shorter segment of the SD became reactivated and a linear orogen was no longer observed. At *α* = 75°, a thrust localized at the distal boundary of the SD for 65% of its length. At *α* = 60°, the reactivated segment of the SD was approximately 42% of its entire length. Where the boundaries of the SD were not reactivated, thrusts followed a trend almost perpendicular to the shortening direction. Different from other experiments, the thrust system developed an arcuate geometry since early stages of shortening (primary curvature). At *α* = 45° the SD had only a minor influence on the orientation and shape of the thrust system. A linear orogen trending perpendicular to the shortening direction developed at approximately half of the model length. Only a short segment of the SD was reactivated in the center of the model, causing a local deviation from the linear trend of the belt.

Model cross sections reveal a thrust system lying on top of a displacement surface developed in the lithospheric mantle (Figures 2b and 6b). Whereas the lower plate of the system is strongly shortened, the upper plate is mostly nondeformed. This underlies that, in such four‐layer lithosphere, the ductile crust strongly decouples the brittle crust from the high‐strength mantle.

Numerous modeling studies investigated the reactivation of pre‐existing structures in oblique compression [e.g., *Bonini et al*., 2012; *Brun and Nalpas*, 1996; *Del Ventisette et al*., 2006; *Panien et al*., 2005]. The results of the experiments presented in this paper show that the angle of obliquity between a tabular stronger domain and the shortening direction determines the length of the reactivated segment. Angles *α* ≥75° allow for 100% reactivation and the development of a linear orogen, while for angles *α* ≤60°, the length of the reactivated segment decreases (Figures 7a and 7b). Similar to the crustal scale models by *Yagupsky et al*. [2008], there is a critical distance of the heterogeneity from the moving wall that determines its reactivation. Moreover, the presented experiments show that the partial reactivation of the crustal heterogeneity is a hitherto undescribed mechanism for the development of an arcuate belt, since curvature develops from the interference between localization in the lithospheric mantle controlled by the wavelength of lithospheric buckling and localization at crustal levels due to the reactivation of the SD. In particular, the arcuate configuration is the result of the intersection of the oblique thrust system parallel to the SD with the thrust system trending perpendicular to the convergence direction (Figure 7a).

Previous analog modeling studies investigated the development of nonrotational arcuate belts (primary arcs) [*Costa and Speranza*, 2003; *Macedo and Marshak*, 1999; *Marques and Cobbold*, 2002]. All these models are crustal‐scale and simulate the presence of lateral variation in (i) crustal strength, like due to seamounts or basement highs [*Costa and Speranza*, 2003; *Macedo and Marshak*, 1999]; (ii) sedimentary basin thickness [*Costa and Speranza*, 2003; *Macedo and Marshak*, 1999]; (iii) topography [*Marques and Cobbold*, 2002]; and (iv) décollement layer [*Costa and Speranza*, 2003]. In the majority of these models the domain of strength variation is smaller than the model width and the curved belt follows its original shape. At variance with these models, in our experiments, the stronger central domain (SD) trends across the entire model width. Consequently, the development of an arcuate belt is controlled by the SD obliquity and its interaction with lithospheric folding and strain localization in the high‐strength lithospheric mantle. This mechanism has not been described in previous analog models of arcuate belts.

### 4.3 A Tentative Application to the Ouachita Orogenic System

**Figure 8:**
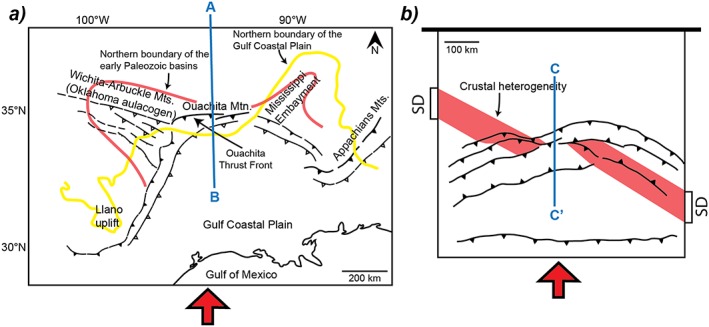
(a) Schematic structural map of the Ouachita orogenic system (inspired mostly from *Arbenz* [1989]). The red arrow indicates the N‐S shortening direction during Carboniferous. The blue line indicated the location of the cross section in Figure 8a. (b) Simplified structural map of *α* = 60° model. Red arrow: shortening direction.

As already mentioned, various mechanisms have been invoked for the origin of arcuate thrust belts such as (i) orogen‐parallel compression [*Johnston et al*., 2013; *Weil et al*., 2013], (ii) slab rollback [*Faccenna et al*., 2004; *Rosenbaum and Lister*, 2004; *Royden*, 1993], (iii) combined thrusting and wrenching [*Brun and Burg*, 1982; *Thomas*, 2011], or (iv) continental indentation [*Macedo and Marshak*, 1999]. In this perspective, the formation of an arcuate thrust system in the experiment carried out with an obliquity *α* = 60° (Figure 5) is of particular interest. This particular model illustrates that the oblique shortening of a continental lithosphere that contains linear high‐strength heterogeneities is a potential mechanism that can lead to the development of large‐scale arcuate thrust belts. The Ouachita system in southern U.S. and northern Mexico (Figure 8a) is an example of arcuate thrust belt that involves the oblique reactivation of pre‐existing rift structures and that, consequently, will be briefly discussed here in the light of our experiment *α* = 60°.

During the breakup of the Rodinia supercontinent, in Late Precambrian‐Early Cambrian, two sets of continental rifts dominantly oriented northwest and northeast (see contours of early Paleozoic basins in Figure 8a) developed in the southern part of Laurentia. Subsequently, the Carboniferous collision between Laurentia and Gondwana continental blocks led to the development of the Ouachita arcuate orogenic system (OAOS). In the central part of the OAOS, whose frontal length is some 2100 km, the Ouachita Mountains correspond along 380 km to an east‐west trending and northward verging thrust belt (Figure 8a). Two lateral and almost linear branches, obliquely connected to the extremities of the Ouachita Mountains, are almost entirely covered by the Mesozoic‐Tertiary sediments of the Gulf Coastal Plain. However, their structure is rather well known, thanks to the dense network of industry seismic and well data. The eastern branch, from the Appalachian Mountains to the Ouachita Mountains, is a continuous thrust belt north‐northwest trending and northeast verging that cuts through the northeast trending early Paleozoic basin of the Mississippi Embayment that was not reactivated during the formation of the OAOS (Figure 8a). To the southeast, the eastern branch is cut by the thrust front of the Appalachian Mountain. The western branch trends almost parallel to the Mississippi Embayment rift with a west‐northwest vergence. It cuts through the west‐northwest trending Wichita‐Arbuckle Mountains that resulted from the reactivation of the northwest trending early Paleozoic rift. The geodynamic interpretation of the OAOS development has been vigorously debated since long and, in particular, the origin of the curvature has been variously interpreted as resulting from (i) the intersection of the two rift trends [*Lowe*, 1985], (ii) the combination of northeast trending rifts and northwest trending transform faults [e.g., *Thomas*, 2011], or (iii) the slab rollback of a secondary subduction zone, moving laterally from the linear Appalachian orogen [*Royden*, 1993].

**Figure 9:**
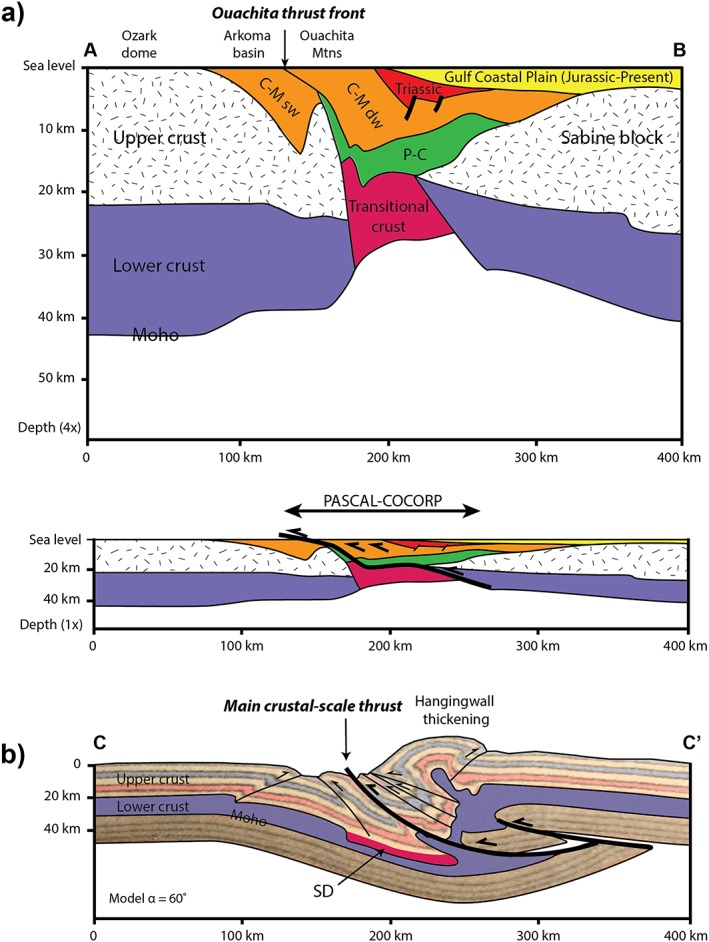
(a) Crustal‐scale section of the Ouachita orogenic system (see location in Figure 8a), (top) with and (bottom) without vertical exaggeration, from deep seismic lines (COCORP [*Nelson et al*., 1982] and PASCAL [*Keller et al*., 1989]) and gravity modeling [*Mickus and Keller*, 1992]. Stratigraphic interpretation (P: Precambrian, C: Cambrian, M: Mississippian, sw: shallow water, dw: deep water) from *Jusczuk* [2002]. (b) Comparison of the Ouachita section (Figure 9a) with the central section of the crust in model *α* = 60° (from section CC′ in Figure 5, see location in Figure 8b). It must be noted that the model section shows a thick crust related to crustal‐scale thrusting, whereas the Ouachita section displays a thin crust at the main thrust zone location due to post‐thrusting erosion and crustal extension related to the Gulf of Mexico opening.

A crustal‐scale section of the Ouachita thrust system (Figure 9a), constructed by using available seismic sections and gravity modeling [*Mickus and Keller*, 1992], shows that the Ouachita orogeny resulted from the convergence between the Laurentia to the north and the Sabine block to the south. During convergence, Precambrian to Mississippian sediments of the Ouachita basin have been thickened by thrusting and folding controlled by several décollement levels (see reviews by *Arbenz* [1989] and *Thomas* [1991]). Dominant northward thrusting started in middle Mississippian and ended in Late Pennsylvanian. Metamorphism is essentially absent, and there is no associated magmatic activity. In other words, the foreland fold‐thrust belt of the Ouachita orogenic system corresponds to the contractional inversion of a rift rather than to a collisional mountain belt following the closure of an oceanic domain, as it has been proposed since long in plate tectonic interpretations [e.g., *Wickham et al*., 1976]. After the Ouachita orogeny, the area has been affected by the extension related to the opening of the Gulf of Mexico, as illustrated in Figure 9a, by a Triassic basin and the Mesozoic to present sedimentary cover of the Gulf Coastal Plain.

Structural studies in the Ouachita Mountains and in its foreland [*Craddock et al*., 1993; *Whitaker and Engelder*, 2006] have documented a north‐south trending regional direction of shortening during the Ouachita orogeny. Therefore, the obliquity between shortening and the Oklahoma aulacogen compares well with our 60° model (Figure 8b). The comparison between the crustal‐scale central section of this model (Figure 9b; from section CC′ in Figure 5) and the Ouachita section (Figure 9a) shows that both cases are controlled by a crustal‐scale thrust zone in the hangingwall of which shortening is located. However, this comparison must take into account that, whereas crustal thickening is well represented in the model, the present‐day section of the Ouachita displays a thin crust in the domain of thrusting (Figure 9a). This difference likely results from erosion of reliefs related to the Ouachita orogeny and from extension and crustal thinning during the opening of the Gulf of Mexico.

The above considerations suggest that the northwest trending Oklahoma aulacogen initially extended southeastward from the Wichita Mountains to the Appalachian Mountains and that only its southeastern part has been significantly reactivated. At variance with our models that contain a single crustal heterogeneity, the Appalachian‐Ouachita region displays two different rift directions that strike NE (Mississippi) and NW (Oklahoma). In this context, the western branch of the arcuate belt that is almost parallel to the Mississippi Embayment rift, with an obliquity of around 30° to the shortening direction (Figure 8a), could likely correspond to a transpressional reactivation, in dominant left‐lateral strike slip, of inherited normal faults [*Brun and Nalpas*, 1996, Figure 4]. A minimum delay of 100 Myr occurred between early Paleozoic rifting and Carboniferous shortening. Therefore, thermal relaxation following rifting was achieved when shortening started. Under such conditions, the rifted domains become stronger than their surrounding nonrifted domains [*England*, 1983]. Consequently, the thin unit of transitional crust that is located at the base of the thrust sedimentary pile (Figure 9a) could have played a role comparable to the strong domain (SD) of our experiments (Figure 8b).

## 5 Conclusions

In summary, our experimental results give valuable insights on the control exerted by oblique reactivation of inherited heterogeneities during lithosphere shortening and more, in particular, on the final plan view geometry (linear versus arcuate) of orogenic systems. The following effects deserved to be mentioned:

1. The presence of a strong domain (SD) controls localization of deformation at its margins.
2. Tectonic inheritance plays a major role in strain localization and controls along‐strike variation in the thrust system.
3. The obliquity angle *α* between the SD and the direction of shortening influences the amount of localization along the SD margins: decreasing with increasing obliquity.
4. When deformation is localized only along a short segment of the SD margins, parts of the SD remain undeformed in the internal or external areas of the orogenic belt.
5. The partial localization along the SD is an alternative mechanism for the initiation of primary arcuate orogens.
6. The Ouachita orogen provides a good example of a curved orogenic belt that likely resulted from the shortening at high angle of a failed continental rift.

## Appendix A Strength Profiles

The strength profiles presented in Figure 1 represent the very initial deformation stage.

For brittle layers (upper crust and sub‐Moho mantle) the maximum resistance in compression has been calculated following *Brun* [2002] as

(A1) $${\left(\sigma_{1}-\sigma_{3}\right)}_{\text{brittle}}=2pgh_{b}$$

where *g* is the acceleration due to gravity (9.81 m/s^2^) and *ρ* and *h_b_* are the density and thickness of the brittle layer, respectively.

The ductile materials used as analog for the lower crust and lithospheric mantle show power law‐type non‐Newtonian behavior (Table 2). Their maximum strength has been calculated according to *Corti et al*. [2004] as

(A2) $${\left(\sigma_{1}-\sigma_{3}\right)}_{\text{viscous}}=2{\left(\frac{\dot{\varepsilon }}{A}\right)}^{1/n}$$

where $\overset{\text{.}}{\varepsilon }$ is the strain rate, calculated as the ratio between velocity and thickness of the ductile layer; *A* is a material parameter function of pressure, temperature, and material properties; and *n* is the stress exponent.

During deformation, layer parallel shearing can develop in the ductile layers. As a consequence, the strain rate is determined as the ratio of convergence velocity and thickness of the ductile layer [*Brun*, 2002]. This gives a high and maximum resistance for the ductile layers. Lower values of differential stress for ductile layers are obtained if the homogeneous strain rate, determined as the ratio of convergence velocity and model length, is considered. Since it is not possible to determine how the strain rate changes during the experiments, we can assume that, during the experiments, the resistance of the ductile layers lies in between the two extremes.
